# Mutation profiling in eight cases of vagal paragangliomas

**DOI:** 10.1186/s12920-020-00763-4

**Published:** 2020-09-18

**Authors:** Anna V. Kudryavtseva, Dmitry V. Kalinin, Vladislav S. Pavlov, Maria V. Savvateeva, Maria S. Fedorova, Elena A. Pudova, Anastasiya A. Kobelyatskaya, Alexander L. Golovyuk, Zulfiya G. Guvatova, George S. Razmakhaev, Tatiana B. Demidova, Sergey A. Simanovsky, Elena N. Slavnova, Andrey А. Poloznikov, Andrey P. Polyakov, Nataliya V. Melnikova, Alexey A. Dmitriev, George S. Krasnov, Anastasiya V. Snezhkina

**Affiliations:** 1 Engelhardt Institute of Molecular Biology, Russian Academy of Sciences, Moscow, Russia; 2 Vishnevsky Institute of Surgery, Ministry of Health of the Russian Federation, Moscow, Russia; 3 National Medical Research Radiological Center, Ministry of Health of the Russian Federation, Moscow, Russia; 4 A. N. Severtsov Institute of Ecology and Evolution, Russian Academy of Sciences, Moscow, Russia

**Keywords:** Vagal paraganglioma, Pathogenic/likely pathogenic mutations, *SDHx* genes, SHD assembly factor-coding genes, Exome, High-throughput sequencing

## Abstract

**Background:**

Vagal paragangliomas (VPGLs) belong to a group of rare head and neck neuroendocrine tumors. VPGLs arise from the vagus nerve and are less common than carotid paragangliomas. Both diagnostics and therapy of the tumors raise significant challenges. Besides, the genetic and molecular mechanisms behind VPGL pathogenesis are poorly understood.

**Methods:**

The collection of VPGLs obtained from 8 patients of Russian population was used in the study. Exome library preparation and high-throughput sequencing of VPGLs were performed using an Illumina technology.

**Results:**

Based on exome analysis, we identified pathogenic/likely pathogenic variants of the *SDHx* genes, frequently mutated in paragangliomas/pheochromocytomas. *SDHB* variants were found in three patients, whereas *SDHD* was mutated in two cases. Moreover, likely pathogenic missense variants were also detected in *SDHAF3* and *SDHAF4* genes encoding for assembly factors for the succinate dehydrogenase (SDH) complex. In a patient, we found a novel variant of the *IDH2* gene that was predicted as pathogenic by a series of algorithms used (such as SIFT, PolyPhen2, FATHMM, MutationTaster, and LRT). Additionally, pathogenic/likely pathogenic variants were determined for several genes, including novel genes and some genes previously reported as associated with different types of tumors.

**Conclusions:**

Results indicate a high heterogeneity among VPGLs, however, it seems that driver events in most cases are associated with mutations in the *SDHx* genes and SDH assembly factor-coding genes that lead to disruptions in the SDH complex.

## Background

Vagal paraganglioma (VPGL) is a neuroendocrine tumor that arises from paraganglia along the course of the vagus nerve (cranial nerve X), the dominant nerve of the parasympathetic division of the autonomic nervous system [1]. The role of vagus paraganglia is not clear enough; they have been proposed to serve a chemoreceptive function and to participate in the immune response to infections [2, 3]. VPGL presents as a painless and slow-growing mass involving the parapharyngeal space [4]. The symptoms of VPGL depend on tumor location. They range from pulsatile tinnitus/ringing in the ear to deficits of cranial nerves (such as hoarseness and dysphagia) and intracranial extension associated with an increased risk of death [5]. VPGL accounts for approximately 13% of all head and neck paragangliomas (HNPGLs) and occurs more frequently in women [6, 7]. Surgical resection is a primary treatment for VPGL, whereas radiation therapy is used in case of malignant and unresectable tumors [8, 9]. VPGL has a lower risk for metastasis compared with carotid paraganglioma (CPGL) [10].

About 30% of HNPGLs develop as inherited tumors [11, 12]. A familial form of HNPGLs predominantly occurs as paraganglioma syndromes and results from mutations in *SDHA*, *SDHB*, *SDHC*, *SDHD,* and *SDHAF2* genes, encoding for succinate dehydrogenase (SDH; mitochondrial complex II) components [11]. Mutations in the *SDHD* gene are the most frequently found in HNPGLs, followed by *SDHB* and *SDHC* mutations [13, 14]. The inheritance pattern for *SDHA,* *SDHB* and *SDHC* is autosomal dominant, whereas for *SDHD* and *SDHAF2* the transmission pattern is consistent with genomic imprinting (with a predominance of paternal transmission) [15]. *SDHAF2* variants rarely present in paragangliomas/pheochromocytomas (PGLs/PCCs) [16, 17]. Familial mutations in *SDHA* have been reported for Leigh syndrome [18] and, most recently, a few have been identified for HNPGLs [19–21]. Mutations in the *VHL*, *TMEM127*, *RET*, *NF1*, and *MAX* genes also harbor the risk for HNPGL development [22, 23]. Additionally, potentially driver somatic/germline variants were identified in several other genes, such as *ARNT*, *BAP1*, *BRAF*, *BRCA1*, *BRCA2*, *CDKN2A*, *CSDE1*, *FGFR3*, *IDH1*, *KIF1B*, *KMT2D*, *MEN1*, *RET*, *JAG1*, *PRDM2, PRDM8*, *SETD2*, *ASPM*, *ZIC*, and *GRIK1* [21, 24, 25].

In this work, we performed whole-exome sequencing of VPGLs from 8 patients. Pathogenic/likely pathogenic mutations were identified and discussed for each individual case and in relation to overall VPGL pathogenesis.

## Methods

### Tumor samples

Formalin-fixed paraffin-embedded (FFPE) VPGL tissues were collected at the Vishnevsky Institute of Surgery, Ministry of Health of the Russian Federation. VPGLs were obtained from 8 patients who did not receive any radiotherapy or chemotherapy before surgery. All patients provided a written informed consent for their participation in the study. This study was approved by the ethics committee from the Vishnevsky Institute of Surgery and performed according to the Declaration of Helsinki (1964).

### DNA isolation, exome library preparation and sequencing

Sections from FFPE tissues were prepared on glass slides and stained with hematoxylin-eosin (H&E). Tumor areas consisting of no less than 80% of tumor cells were chosen in unstained sections according to reference H&E-stained slides and transferred into microcentrifuge tubes. DNA was isolated from 9 different tumor tissues using the High Pure FFPET DNA Isolation Kit (Roche, Switzerland). Among the 9 tumors, 2 belonged to 1 patient (Patient 2) and samples were obtained from different FFPE blocks. DNA concentration was measured on a Qubit 2.0 fluorometer (Thermo Fisher Scientific, USA). DNA quality was tested using an Agilent 2100 Bioanalyzer (Agilent Technologies, USA) and quantitative PCR (qPCR) was performed with a QuantumDNA Kit (Evrogen, Russia).

In total, 9 exome libraries were prepared using the Nextera Rapid Capture Exome Kit (Illumina, USA) according to the manufacturer’s instructions. High-output sequencing was performed on a NextSeq 500 System (Illumina) with paired-end 76 × 2 cycles. At least 300× coverage for each sample was obtained. Raw sequence data were deposited into the NCBI Sequence Read Archive (SRA) under accession number PRJNA561073.

### Bioinformatics analysis

Illumina paired-end reads were subjected to quality control with FastQC. Then, reads were trimmed and adapters removed using Trimmomatic [26]. Next, reads were mapped to the reference human genome GRCh37.75/hg19 (Ensembl) using BWA [27]. Derived BAM files were sorted with SAMtools [28] and processed with Picard tools (files were reordered and assigned to group names, whereas duplicated reads were marked; https://broadinstitute.github.io/picard/index.html). Besides, we performed base quality score recalibration using GATK4 (v. 4.1.2) and dbSNP (build 144) [29]. Variant calling was performed with GATK 4.1.2 HaplotypeCaller [29]. We turned on StrandBiasBySample, StrandOddsRatio, and BaseQualityRankSumTest annotations in order to exclude false positives. Additionally, we excluded SNVs in polyN motifs, such as GGGTG > GGGGG and CCCCG > CCCCC, which are frequently missequenced by Illumina NextSeq 500 system.

The derived list of variants was annotated using Annovar [30]. For this part of the analysis, we included allele population frequencies databases (gnomAD, 1000 Genomes Project, Kaviar, ESP6500, and ExAC), dbSNP, ClinVar, and COSMIC annotations, localization in a protein domain (Interpro), and site conservation data (phastCons and PhyloP). Additionally, we included annotations by DANN, CADD, SIFT, PolyPhen-2, FATHMM, MutationAssessor, MutationTaster, LRT, PROVEAN, M-CAP, Meta-SVM, and MetaLR in order to assess the pathogenicity of the variants. Finally, the “pathogenicity score” was calculated based on the population frequency, site conservation, and the summary weighted score across several prediction algorithms. Additionally, obtained “pathogenicity scores” for variants annotated as pathogenic by the ClinVar or contained in the COSMIC v70 database were increased.

## Results and discussion

We performed whole-exome sequencing of VPGLs from 8 patients. Normal tissues from the same patients were not available due to the fact that samples were originally collected as archive material. In VPGLs, we identified pathogenic/likely pathogenic variants of genes that were previously shown to be associated with PGLs/PCCs: *VHL, SDHx, NF1, RET, HRAS, KRAS, EPAS1* (*HIF2A*)*, ATRX, CSDE1, BRAF, FGFR1, FGFR2, FGFR3, FGFR4, FGFRL1, SETD2, ARNT, TP53, TP53BP1, TP53BP2, TP53I13, KMT2D, BAP1, IDH1, IDH2, SDHAF1, SDHAP2, FH, EGLN1, MDH2, TMEM127, MAX, KIF1B, MEN1, GDNF, GNAS, CDKN2A, BRCA1,* and *BRCA2* [21] as well as in other groups of genes (Additional file 1). Pathogenicity of the variants was predicted based on several resources, including prediction tools, population frequency databases, open sources on the clinical significance of variants, and algorithms for estimation of sequence conservation scores. The description of each case in detail is presented below. Besides, Table 1 shows the main clinic pathological characteristics of the patients. All patients were female.

**Table 1:** Clinic pathologic characteristics of the patients with VPGLs

Patient	Age, yr	Tumor localization (left/right side of the neck)	*SDHx* and *SDHAF1–4* mutations
1	70	Left	No
2	52	Right	*SDHB*
3	41	Left	*SDHAF3* *SDHAF4*
4	51	Left	No
5	50	Right	*SDHB* *SDHAF4*
6^a^	50	Right	*SDHD*
7	26	Left	*SDHB*
8	68	Left	*SDHD*

^a^ Patient with multiple paragangliomas

### Patient 1

A 70-year-old female presented with a volumetric formation on the left submandibular region with a concomitant diagnosis of arterial hypertension. Ultrasound study and computed tomography (CT) revealed a tumor 28 × 21 × 21 mm in size at the left carotid bifurcation. At surgery, the tumor originating from the vagus nerve was excised. Histological examination of postoperative material confirmed a paraganglioma of solid trabecular structure.

Exome analysis of the tumor revealed no variants in the potentially causative genes for PGLs/PCCs. However, we found likely pathogenic variants characterized by high “pathogenicity scores” as well as by frameshift mutations in several tumor-associated genes, such as *CYSLTR2*, *GPX2*, *ENPP7*, *CD34*, *UBA7*, and others (Additional file 1).

For the *UTS2R* gene, we identified a likely pathogenic variant, NM_018949: c.C313A, p.P105T (chr17: 80332513, rs140576840), that had not been reported in the ClinVar or COSMIC databases before. *UTS2R* encodes for urotensin II receptor (G-protein coupled receptor 14, GPR14), a protein that has been shown to be involved in PCC pathogenesis. It has been shown that the incubation of human pheochromocytoma cells (primary culture) with urotensin II promotes cell proliferation [31]. Moreover, *UTS2R* mRNA levels are decreased in PCC [32].

A likely pathogenic variant, NM_004804: c.C296T, p.T99I (chr2: 96933370, rs780418079), was also determined for the *CIAO1* gene. This variant had not been described neither in the ClinVar nor in the COSMIC databases. The *CIAO1* gene encodes for the cytosolic iron-sulfur assembly component 1 participating in energy metabolism. Alterations in *CIAO1* are associated with hereditary PGL/PCC syndromes according to the MalaCards database (https://www.malacards.org/).

The likely pathogenic missense variant NM_017558: c.G1352C, p.R451P (chr16: 71127814, rs7200485) of the *HYDIN* gene was also identified in this VPGL. Nevertheless, the variant had not been described in the ClinVar or COSMIC databases. Noteworthy, we have previously shown that *HYDIN* is characterized by a high mutation rate in CPGL and could be involved in its pathogenesis [33].

Additionally, we found several likely pathogenic variants reported in the COSMIC database for the following genes: *CD109* (COSM1621920; liver and lung cancer), *CYSLTR2* (COSM1367341; colon cancer), *DENND6B* (COSM1035425; endometrial cancer), and *OR9I1* (COSM385271; lung cancer).

### Patient 2

A 52-year-old female, complaining from arterial hypertension, was diagnosed with a right-sided neck mass. After a year, an excisional biopsy was performed and, a year later, several complications appeared: a deviation of the right side of the tongue, hoarseness, troublesome swallowing, and right papilla atrophy. A year after the appearance of these complications, the patient was hospitalized for surgery. It was then when the presence of a VPGL on the right side of the neck, in the area of the carotid artery bifurcation, was determined by color duplex scanning. It was also found that regional lymph nodes were increased in size. A right-sided hypervascularized VPGL, 72 × 27 × 27 mm in size, spreading from the jugular hole to the carotid bifurcation, involving both the carotid artery and the hyoid nerve, was revealed by CT scans with 3D reconstruction, confirming the diagnosis. An ultrasound study of internal organs showed a moderately pronounced bilateral nephropathy and diffuse changes in pancreatic tissue. At surgery, it was found that the tumor originated from the vagus nerve and reached the base of the skull involving the hyoid nerve. The tumor was removed along with resection of both the vagus and hyoid nerves, the outer carotid artery, and regional lymph nodes. Histological examination revealed a typical organoid paraganglioma of alveolar and trabecular structure without regional lymph node metastasis. The patient was extubated in the operating room; however, the early postoperative period was complicated by cardiopulmonary failure, followed by aspiration pneumonia. The patient died 9 days after surgery.

For this patient, we performed whole-exome sequencing of two tumor samples from different FFPE blocks to validate sequencing data. Results obtained from both samples were in concordance with each other (Additional file 1). We detected a likely pathogenic missense variant in the *SDHB* gene, NM_003000: c.A307G, p.M103V (chr1: 17355211, rs140178341). This variant is described in the ClinVar database as a variant with uncertain clinical significance. Its presence was previously detected for Cowden syndrome 2 (a hereditary cancer-predisposing syndrome), gastrointestinal stromal tumor, PGLs/PCCs, and PGL 4 syndrome, during clinical testing. Additionally, two novel frameshift mutations were found in *SDHB*, NM_003000: c.308_309insTAAG, p.M103fs (chr1: 17355209) and NM_003000: c.304_305insATGAT, p.A102fs (chr1: 17355213). A likely pathogenic missense variant was also observed in *ARNT*, NM_001197325: c.C1834T, p.R612C (chr1: 150788806, rs778430234); a variant that had not been previously reported, neither in the ClinVar nor in the COSMIC databases. However, likely pathogenic variants in the *ARNT* gene were earlier determined in CPGLs [21].

According to several prediction algorithms, likely pathogenic variants as well as frameshift and stop-gain mutations were also identified in other genes (Additional file 1). One of these distinctive genes is a novel likely pathogenic variant for *RXFP1*, NM_021634: c.G172A, p.D58N (chr4: 159493972). This gene encodes for the relaxin family peptide receptor 1, a protein related to RLN2/RXFP1 signaling that has been shown to mediate tumor cell motility and invasion in breast, thyroid, prostate, and endometrial cancers [34–38]. The induction of the CTRP8-RXFP1 ligand-receptor system increases the migration of glioblastoma cells [39]. Moreover, RXFP1 participates in cancer chemoresistance [40].

A likely pathogenic variant NM_024776: c.C2908T, p.R970C (crh15: 77471361, rs117879553) in the *PEAK1* gene was also revealed in this patient’s VPGL. The variant had not been described in the ClinVar or COSMIC databases. However, numerous studies have reported the involvement of *PEAK1* in tumorigenesis [41–45].

The *SLIT2* gene encodes for the slit homolog 2 protein, a ligand of the ROBO immunoglobulin receptor family. The SLIT/ROBO pathway plays an important role in axon guidance and neuronal precursor cell migration as well as in the regulation of angiogenesis [46]. We determined a likely pathogenic variant NM_004787: c.A934G, p.I312V (chr4: 20512137, rs139850475) for this gene. According to data from the literature, the SLIT/ROBO pathway has a dual effect in tumor growth and progression. On the one hand, SLIT/ROBO promotes tumor cell proliferation and migration [47]. On the other hand, it has been reported that *SLIT2* is frequently inactivated in gliomas by hypermethylation at the CpG island within its promoter region and can act as a tumor suppressor gene [48].

A likely pathogenic variant was also found in the *AMPH* gene, NM_001635: c.A2024G, p.Q675R (chr7: 38424483, rs147019216T). *AMPH* encodes for amphiphysin 1, localized in nerve terminals of mature neurons and participates in clathrin-mediated endocytosis [49, 50]. Noteworthy, the expression of amphiphysin 1 was detected in patients with stiff-person syndrome associated with breast and lung cancer, and melanoma [51]. Additionally, *AMPH* knockdown reduces apoptosis and promotes cell cycle progression and migration of breast cancer cells [52].

In the *HAPLN3* gene, we also identified a likely pathogenic variant, NM_178232: c.G316A, p.G106R (chr15: 89424765, rs140982817), described in the COSMIC database as associated with lung and caecum cancer (COSM1375297).

### Patient 3

A 41-year-old female was examined for the presence of neoplasm on the left side of the neck. It was noticed that the neoplasm was detected 10 years before the examination and grew fast for the last five years only. CT analysis showed that the tumor was located at the bifurcation of the common carotid artery (CCA) (on the posterior surface of the formation) from the thyroid upper pole level. The presence of a homogeneous structure; a soft tissue density mass with smooth, sufficiently clear hilly contours, measuring 70 × 47 × 42 mm, was determined. The tumor belonged to the anterior medial surface of the outer carotid artery (OCA) (pushing the vessel laterally) and was located between the external and internal carotid arteries. The jugular vein was also involved by the mass. The upper pole of the formation was 40 mm lower than the base of the skull. The tumor was removed after embolization to reduce blood loss. Histological testing of the operation material showed a mature paraganglioma with an alveolar and trabecular structure without any symptom of lymph node metastasis.

Exome analysis of the VPGL revealed the existence of likely pathogenic missense variants of both the *SDHAF3* and *SDHAF4* genes: NM_020186: c.T157C, p.F53L (chr7: 96747192, rs62624461) and NM_145267: c.C223T, p.P75S (chr6: 71298323, rs146446063), respectively (Additional file 1). These variants had not been reported in the ClinVar or COSMIC databases before. Both genes encode for SDH assembly factors and play a critical role in SDH stability and activity. Previously, we found a likely pathogenic missense variant of *SDHAF3* from CPGL (not published). A variant of this gene, which may probably damage the protein function (according to the in silico tools used for analysis), was determined in familial and sporadic PGLs/PCCs [53].

For VPGL, we revealed several likely pathogenic variants of the *COL19A1* (COSM1235991; related to plasma cell myeloma and lung cancer), *SEC31B* (COSM913945; involved in endometrial and colorectal cancer), *THSD7B* (COSM247830; associated with prostate cancer), and *TIAM1* (COSM1566097, COSM1566096; related to rectum cancer) genes, which have been previously described in the COSMIC database.

Additionally, we identified likely pathogenic variants of *CRNN*, *CYP4B1*, *ELL*, *ENPP7*, *FAT2*, *NCOR2*, *SART1*, *TIAM1*, *VIM*, and other genes involved in tumorigenesis according to literature data.

### Patient 4

A 51-year-old female patient was examined for complaints of swelling on the left side of the neck, hoarseness, morning headaches, weakness, and poor sleep. Enlarged lymph nodes were bothering her since a sore throat presented two years before. Duplex scanning (DS) and CT revealed a solid tumor 26 × 19 × 29 mm in size on the left side of the neck at the submandibular region, lateral to the internal carotid artery (ICA) and the OCA; the tumor bordered the arteries. During surgery, it was found that both the CCA bifurcation and initial parts of the ICA and OCA were involved in the tumor. A thickening of the trunk of the vagus nerve was also detected; it was found that the tumor originated precisely from the nerve. Besides, the upper pole of the tumor was reaching the base of the skull and the proximal part of the hypoglossal nerve was intimately soldered to the tumor capsule for as long as 5 mm. The glossopharyngeal nerve, adjacent to the upper pole of the tumor, was also intimately associated to the capsule of the tumor for as long as 5 to 7 mm. The tumor was resected along with the proximal end of the vagus nerve. Histological examination revealed a mature paraganglioma with alveolar and trabecular structure.

In the VPGL of this patient, we found no pathogenic/likely pathogenic variants for any of the PGL/PCC potentially causative genes, including *SDHx* (Additional file 1). Nevertheless, for several genes, we identified likely pathogenic variants reported in the COSMIC database and associated with different types of cancer: *ACAD11* (COSM3695823; colon cancer), *MTNR1B* (COSM1561913; rectum, lung, liver, and colorectal cancer), *NTF4* (COSM3404440; glioma and rhabdomyosarcoma), *PATZ1* (COSM273076; acute lymphoblastic leukaemia and colon cancer), *SAMD9L* (COSM3784101; prostate cancer), and *TRIM16* (COSM401683; lung cancer).

A likely pathogenic variant was also revealed for the *AMBRA1* gene, involved in cancer development and chemotherapy resistance [54]. Several other genes with likely pathogenic variants that were previously shown to participate in tumorigenesis were also found within this patient’s exome. For example, the *CHST4* gene, which is associated with colon mucinous adenocarcinoma (MalaCards database); *CLCA4*, a tumor suppressor gene encoding for the calcium-activated chloride channel A4 that can inhibit tumor cell proliferation, migration, and invasion [55, 56]; *ESR1*, characterized by mutations responsible for the resistance to endocrine therapy in breast cancer [57]; *P2RY1*, involved in the regulation of multidrug chemoresistance of bladder cancer cells [58]; and *PATZ1*, which acts as a tumor suppressor gene in thyroid cancer and is associated with the development of testicular tumors, sarcoma, and lung cancer [59–62]. Furthermore, we found alterations in the *PRKCI* gene related to many tumors [63–65] and *WIF1* hypermethylation, frequently observed in cancer cells [66–69].

### Patient 5

A 50-year-old female patient troubled by intense dizziness for several days, mainly during spring and autumn, for the last ten years. The patient self-detected a mass in the right submandibular region. After visualization by ultrasound study, a cyst of the neck was suspected and an attempt to remove it was unsuccessful, leading to suspicion of a paraganglioma. DS showed a 56 × 34 mm body in the CCA bifurcation, involving the initial parts of both the OCA and ICA. The patient was hospitalized for follow-up and surgical treatment. Further studies by CT imaging revealed a neck mass 45 × 32 × 47 mm in size next to the ICA and the OCA, whereas an angiography showed a VPGL on the right side of the neck. The lower pole of the neoplasm was located 10 mm higher than the carotid bifurcation, whilst the upper pole was 30 mm lower than the base of the skull. As an attempt to reduce blood loss, an embolization was performed first, followed by paraganglioma excision with right ICA resection. Histological analysis of the tissues revealed a mature paraganglioma with alveolar and trabecular structure.

Exome analysis of the VPGL revealed likely pathogenic variants in several PGL/PCC potentially causative genes: *SDHB*, *SDHAF4*, *FGFRL1*, and *IDH2* (Additional file 1). A splice acceptor mutation identified in the *SDHB* gene (chr1: 17350571, rs786201161) was described as a germline pathogenic variant in the ClinVar database. This variant has been previously reported in association with an SDHB-related disorder, hereditary cancer-predisposing syndrome, gastrointestinal stromal tumor, and hereditary PGL/PCC syndromes [70–72]. The mutation destroys the canonical splice acceptor site within intron 5 of the *SDHB* gene and is expected to cause abnormal gene splicing, likely resulting in an absent or disrupted protein product. For *SDHAF4*, we found a novel likely pathogenic missense mutation NM_145267: c.C223T, p.P75S (chr6: 71298323, rs146446063). The likely pathogenic missense mutation identified in the *FGFRL1* gene, NM_001004356: c.C442T, p.R148C (chr4: 1017613, rs201696860), has not been previously reported in the ClinVar database; however, it was detected in lung adenocarcinoma and described in the COSMIC database (COSM119740). Finally, in the *IDH2* gene, we found a novel mutation NM_001289910: c.T998C, p.L333P (chr15: 90628257) that was predicted as a likely pathogenic variant according to all the prediction algorithms used.

For this VPGL, we also determined likely pathogenic variants in a few genes that were reported in the COSMIC database and associated with different types of cancer: *ATP2A1* (COSM1184049; colon cancer), *ACAD11* (COSM3695823; colon cancer), *ERCC4* (COSM967199; endometrial, colorectal, and papillary thyroid cancer), and *VPS13B* (COSM3698729, COSM3698730; colon cancer and lung bronchioloalveolar adenocarcinoma). Moreover, likely pathogenic variants were identified in genes reported as tumor-associated ones according to the literature; for example, in *CD248*, *LGR5*, *LRP1B*, *PBRM1*, and *PTPA*.

### Patient 6

A 50-year-old female, complaining from arterial hypertension was diagnosed with neck masses on both sides of the body. Both CT and ultrasound study revealed the presence of heterogeneous and hypervascular tumors at the carotid bifurcation areas: a solid neoplasia on the left side and a two-nodal tumor on the right side of the neck. The left tumor, 25 × 23 × 17 mm in size, was resected. Then, the patient was discharged with a planned re-hospitalization to remove the other tumor. After a year, the tumor, 35 × 20 mm in size, located directly in the carotid artery bifurcation on the right side of the neck, and one more tumor, 60 × 20 mm in size, originating in the vagus nerve, were removed. During the surgery, the lymph node from above the carotid artery bifurcation was also resected. All tumors and lymph nodes were subjected to histological examination. Paragangliomas with no lymph node metastases were revealed.

Using whole-exome sequencing, we found a pathogenic germline mutation in the *SDHD* gene, NM_003002: c.A305G, p.H102R (chr11: 111959726, rs104894302) (Additional file 1). This variant is described in the ClinVar database as a likely pathogenic germline one and has been found in malignant paraganglioma [73]. At the same position in the *SDHD* gene (codon 102), a sequence change leading to an amino acid substitution of a histidine for either a leucine, a proline, or a tyrosine was also observed in individuals with paragangliomas [74–76]. This indicates that this histidine residue plays a critical role in SDHD protein function.

In VPGL, we also identified likely pathogenic variants reported in the COSMIC database for the following genes: *ABCB4* (COSM1092579; endometrial and colorectal cancer), *LHX8* (COSM1560500; rectum and stomach cancer), *DAPK2* (COSM1678527; clear cell renal cell carcinoma), *STARD10* (COSM1475880; breast cancer), and *ZNF599* (COSM328007; pancreas cancer).

Additionally, likely pathogenic variants were determined in a number of genes that have been shown to be involved in oncogenic processes. For example, the *ARPC5* gene encodes for the actin-related protein 2/3 complex subunit 5 that can affect tumor cell growth, migration, and invasion [77, 78]. *MAP 2* has an essential function in neurogenesis; however, alterations in this gene’s regulation occur in melanoma, glioma, and oral and gastric cancers [79–82]. The proto-oncogene *ROS1* belongs to the subfamily of tyrosine kinase insulin receptor genes; *ROS1* fusions were found in a variety of tumors [83]. The protein product of the *SETD3* gene participates in lysine degradation and chromatin organization; alterations in this gene are associated with renal cell carcinoma according to the MalaCards database.

### Patient 7

A 26-year-old female went to a medical facility with a blocked brachial plexus and pain both in the arm and the suprascapular region. The pain felt in the left half of the neck and head, the left arm, and the suprascapular region had bothered her for about a year. Moreover, dizziness and headaches had been present since adolescence. CT and magnetic resonance imaging revealed an unorganized swelling of the neck on the left side, 25 × 20 mm in size. An US showed that the neoplasm was a hypervascular tumor with its borders on the subclavian artery. A Matas test made to the outpatient was positive: Clamping the CCA caused retrograde blood flow in the supratrochlear artery. During surgery, the tumor was separated from the lateral wall of the СCA and the upper wall of the subclavian artery and excised as a single conglomerate. The tumor, presumably coming from the sympathetic ganglion, was considered as a vagal paraganglioma. Using histological examination, a paraganglioma with an alveolar structure was observed.

In this patient, we found a pathogenic stop-gain mutation NM_003000: c.C79T, p.R27X (chr1: 17371377, rs74315369) in the *SDHB* gene. This mutation is described in the ClinVar database as a germline variant associated with PGLs/PCCs, hereditary cancer-predisposing syndrome, and early onset renal cell cancer [84–86] (Additional file 1). This mutation results in a premature stop codon in exon 2, disrupting SDHB expression and function.

In this patient’s VPGL, we first identified likely pathogenic variants in a number of genes that have previously been found in other tumors according to the COSMIC database: *VSIG10* (COSM935687; endometrial cancer), *MRGPRX1* (COSM3687222; colon and lung cancer), *DUSP27* (COSM144660; diffuse large B cell lymphoma), and *C10orf88* (COSM3686556; colon cancer). Moreover, tumor-related alterations were also found for several other genes (*ABCA3*, *ABI3*, *ATG4C*, *BLZF1*, *BMP10*, *CHD5*, *CNKSR1*, *CRB3*, *DBH*, *FAM83A*, *FAT2*, *GNA13*, *KIF26B*, *MC1R*, *NR4A1*, *NUP155*, *PCM1*, *SGK2*, and *UBR5*), in which likely pathogenic variants were observed for VPGLs.

### Patient 8

A 68-year-old female patient had constant episodes of dizziness, headaches, sore throat, and lump feeling in the neck and was examined for a complaint of neoplasms on the left side of the neck. The patient suffered from a long history of arterial hypertension and underwent a surgery for the removal of a thyroid goiter (1990) due to a tumor of the anterior mediastinum (intrathoracic goiter). At surgery, a hypervascular tumor, 60 mm in diameter, originating from the left vagus nerve, was resected. It was found 30 mm below the bifurcation of the left CCA and located mainly behind it and behind the ICA and OCA, reaching the base of the skull, involving the ICA in its upper part (the artery was flattened on the upper pole of the tumor). The hyoid and the glossopharyngeal nerves were also removed. Along the sternocleidomastoid muscle, two enlarged lymph nodes were resected and subjected to histological examination. A paraganglioma of alveolar and trabecular histological structure without lymph node metastasis was determined.

Using the whole exome-sequencing, a missense mutation in the *SDHD* gene, NM_003002: c.A305G, p.H102R (chr11: 111959726, rs104894302), was determined in the VPGL (Additional file 1). This was the same mutation that was identified in Patient 6 (described above).

According to the COSMIC database, we found likely pathogenic variants that were earlier detected in other malignancies: *TLL1* (COSM1671424; colorectal cancer), *CDH8* (COSM972084; endometrial, pancreatic, and skin cancer), *ANAPC5* (COSM936016; endometrial cancer), *ACAN* (COSM3690591, COSM3690590; colon cancer), *C7orf72* (COSM3698459; colon cancer), and *ZNF599* (COSM328007; pancreatic cancer).

Additionally, likely pathogenic variants were revealed in well-known tumor-associated genes. For example, variant NM_004996: c.C3901T, p.R1301C (chr16: 16225727, rs201533167) was detected in the *ABCC1* gene; this variant seems to be pathogenic according to all the prediction tools used for analysis. ABCC1 belongs to the superfamily of ATP-binding cassette (ABC) transporters and plays an important role in the response to cancer chemotherapy [87]. The *ALDH1L1* gene, in which we found the likely pathogenic variant NM_012190: c.G68A, p.G23D (chr3: 125879755, rs143122118), is a potential tumor suppressor gene, encoding for a protein involved in folate metabolism [88]. A likely pathogenic variant NM_007194: c.T1091C, p.I364T (chr22: 29092893, rs774179198) was revealed in the *CHEK2* gene, which is commonly mutated in hereditary cancer [89]. In *COL4A1*, a variant NM_001845: c.G4442A, p.R1481Q (chr13: 110814597, rs376607450) was observed. *COL4A1* encodes for the type IV collagen alpha protein that can contribute to tumor cell migration and invasiveness [90]. In the *MADD* gene, a novel stop-gain variant NM_003682: c.C2077T, p.Q693X (chr11: 47306036) was found. This gene is involved in tumor cell growth and metastasis [91]. In this patient’s VPGL, we also observed a variant NM_015641: c.G1123A, p.V375M (chr7: 115897393, rs202205760) in *TES*, a tumor suppressor gene involved in the regulation of tumorigenesis, angiogenesis, and metastasis [92]. Additionally, a likely pathogenic variant NM_017672: c.C1337G, p.A446G (chr15: 50916466) in the *TRPM7* gene was found. This gene encodes for a protein that acts as both an ion channel and a serine/threonine-protein kinase. A few studies considered *TRPM7* as a potential biomarker and therapeutic target for many tumors, including pancreatic, breast, gastric, and ovarian cancers, glioblastoma, T cell leukemia, and hypopharyngeal squamous cell carcinoma [93].

## Conclusions

In this study, exome data of 8 female patients with VPGL were analyzed. In 6 of them, we revealed the existence of pathogenic/likely pathogenic variants of genes encoding for components of the SDH complex (*SDHB*, *SDHD*, *SDHAF3*, and *SDHAF4*), confirming their high mutation frequency not only in paragangliomas and pheochromocytomas but also in VPGLs as distinct tumor types. Moreover, likely pathogenic variants were identified in different tumor-associated genes as well as variants that have previously been found in common tumors, such as lung, colorectal, endometrial, and renal cancers. Our results indicate a high heterogeneity among VPGLs that is supported by a wide number of identified pathogenic/likely pathogenic variants in different genes (besides mutations in *SDHx/SDHAF1–4* genes) in each patient. Obtained data contribute to a better understanding of involvement of specific genes and possible molecular mechanisms in the pathogenesis of this kind of tumor.

## Supplementary information

**Additional file 1.** Pathogenic/likely pathogenic variants identified in the patients with vagal paragangliomas. (XLS 339 kb)

## Data Availability

All data generated or analyzed in this study are included in the published article. The sequence data are available in the NCBI SRA under the accession number PRJNA561073.

## Abbreviations

PGL: Paraganglioma
PCC: Pheochromocytoma
HNPGL: Head and neck paraganglioma
VPGL: Vagal paraganglioma
CPGL: Carotid paraganglioma
CT: Computed tomography
DS: Duplex scanning
MRI: Magnetic resonance imaging
CCA: Common carotid artery
ICA: Internal carotid artery
OCA: Outer carotid artery
SDH: Succinate dehydrogenase
